# Effects of Fruit and Vegetable Consumption on Risk of Asthma, Wheezing and Immune Responses: A Systematic Review and Meta-Analysis

**DOI:** 10.3390/nu9040341

**Published:** 2017-03-29

**Authors:** Banafshe Hosseini, Bronwyn S. Berthon, Peter Wark, Lisa G. Wood

**Affiliations:** 1 School of Biomedical Sciences and Pharmacy, Faculty of Health and Medicine, University of Newcastle, Callaghan, NSW 2308, Australia; b.hosseini.bh@gmail.com (B.H.); bronwyn.berthon@newcastle.edu.au (B.S.B.); 2 Centre for Healthy Lungs, Hunter Medical Research Institute, Newcastle, NSW 2308, Australia; Peter.Wark@hnehealth.nsw.gov.au

**Keywords:** fruit, vegetable, antioxidant, asthma, wheezing, immune response

## Abstract

Evidence suggests that reduced intake of fruit and vegetables may play a critical role in the development of asthma and allergies. The present review aimed to summarize the evidence for the association between fruit and vegetable intake, risk of asthma/wheeze and immune responses. Databases including PubMed, Cochrane, CINAHL and EMBASE were searched up to June 2016. Studies that investigated the effects of fruit and vegetable intake on risk of asthma/wheeze and immune responses were considered eligible (*n* = 58). Studies used cross-sectional (*n* = 30), cohort (*n* = 13), case-control (*n* = 8) and experimental (*n* = 7) designs. Most of the studies (*n* = 30) reported beneficial associations of fruit and vegetable consumption with risk of asthma and/or respiratory function, while eight studies found no significant relationship. Some studies (*n* = 20) reported mixed results, as they found a negative association between fruit only or vegetable only, and asthma. In addition, the meta-analyses in both adults and children showed inverse associations between fruit intake and risk of prevalent wheeze and asthma severity (*p* < 0.05). Likewise, vegetable intake was negatively associated with risk of prevalent asthma (*p* < 0.05). Seven studies examined immune responses in relation to fruit and vegetable intake in asthma, with *n* = 6 showing a protective effect against either systemic or airway inflammation. Fruit and vegetable consumption appears to be protective against asthma.

## 1. Introduction

Asthma is a chronic inflammatory lung disease, associated with airway constriction, inflammation, bronchial hyper-responsiveness (BHR), as well as respiratory symptoms such as coughing, wheezing, dyspnoea and chest tightness. The rise in incidence, prevalence and related medical and economic costs of asthma across all age groups is a public health concern [1]. In Australia, one in every 10 adults has asthma. It has been estimated that currently about 300 million people suffer from asthma worldwide, with 250,000 annual deaths related to the disease. It is also estimated that the prevalence of asthma will grow by more than 100 million by 2025 [2]. Asthma is the consequence of complicated interactions between genetics and environmental factors. In genetically susceptible people, such interactions can lead to the development of airway inflammation, atopy and/or BHR [3]. Environmental factors including tobacco smoke, allergen exposure, pollen, mites, air pollution, chemical sprays, high ozone levels, broad-spectrum antibiotic usage during the first years of life, small size at birth, having few siblings, as well as respiratory infections such as Rhinovirus (RV) can play a major role in developing asthma exacerbations [4,5]. The considerable morbidity related to asthma may be ameliorated by addressing modifiable risk factors such as diet [6]. It has been suggested that the increased prevalence of asthma in recent decades may be associated with changes in dietary habits since the 1950s—particularly, deficiency in dietary antioxidants [7]. The Western diet has shifted towards less fruit and vegetables, and high intakes of convenience foods that are low in fibre and antioxidants and rich in saturated fats [8,9].

Oxidative stress plays a major role in the pathophysiology of asthma, due to chronic activation of airway inflammatory cells [10]. There is ample evidence that oxidative stress can have various deleterious effects on airway function, including airway smooth muscle contraction, induction of BHR, mucus hypersecretion, epithelial shedding and vascular exudation [11,12]. Moreover, reactive oxygen species (ROS) can activate transcription factor nuclear factor-kappa B (NF-κB), which results in a cascade of events involving upregulation of the transcription of various inflammatory cytokine genes, such as interleukin-6 (IL-6) and eventually influx and degranulation of airway neutrophils [8]. Fresh fruit and vegetables provide rich sources of antioxidants and other biologically active substances (such as flavonoids, isoflavonoids and polyphenolic compounds) [12]. Studies have shown that diets with low average consumption of fruit and vegetables play a major role in the development of allergic diseases [1,7], and may augment oxidative stress in asthma [13]. Antioxidants can reduce airway inflammation via protecting the airways against oxidants by both endogenous (activated inflammatory cells) and exogenous (such as air pollution, cigarette smoke) sources [7]. Moreover, dietary antioxidants present in fruit and vegetables can scavenge ROS, and thus inhibit NFκB-mediated inflammation, while diets low in antioxidants have reduced capacity to respond to oxidative stress [8].

Currently available asthma medications, such as glucocorticoids, are ineffective in some cases such as viral-induced exacerbations [14]; and prolonged treatment with these therapeutic agents can result in adverse effects, such as pneumonia, cataracts, and osteoporosis [15]. Therefore, non-pharmacological interventions are required to reduce the burden of asthma in both adults and children. Understanding the roles of dietary nutrients in asthma and asthma-related complications may help in the management of this chronic inflammatory disease. Hence, a systematic review of the intake of fruits and vegetables and their effects on immune responses and asthma risk is of interest. This paper aimed to describe studies investigating the effects of fruit and vegetable consumption on risk of asthma and wheezing and immune responses (including immune responses to virus infection and inflammation) in asthma and wheezing.

## 2. Methods

### 2.1. Search Strategy

**Figure 1:**
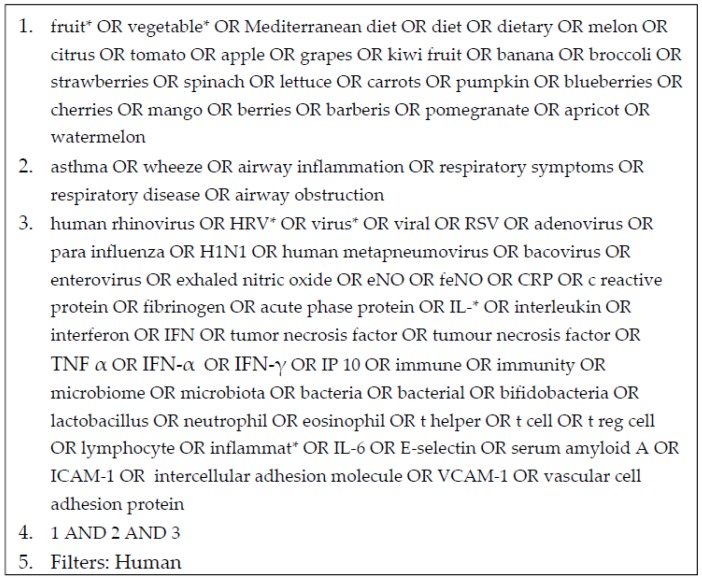
Example of search strategy using PubMed for studies investigating the effects of fruit and vegetable consumption on immune responses (including immune responses to virus infection, and inflammation) and clinical outcomes in asthma.

PubMed, Cochrane, CINAHL and EMBASE databases were included in the literature search, which was conducted in June 2016, including all previously published articles. Studies were limited to humans with no language restrictions. Additional studies were identified by hand searching references from the identified studies. See Figure 1 for an example of the search strategy.

### 2.2. Study Selection

Only original studies with the following designs were included: randomized controlled trials, quasi-experimental studies, cohort studies, case-control studies, before and after studies, and cross sectional studies. Case studies, case reports, animal studies, opinion papers, in vitro studies and conference abstracts were excluded. Review articles were collected for the purposes of reviewing the reference list and did not contribute to the final number of included studies. The target study population was human of all age, gender or ethnicity, with asthma, wheeze, airway inflammation or other related respiratory symptoms. The exposure of interest was intake of whole or extracted fruit and vegetables. The study outcome measures were respiratory virus infection including human rhinovirus, influenza virus, corona virus and adenovirus; markers of systemic inflammation such as ILs, C-reactive protein, tumour necrosis factor-α and intercellular adhesion molecule 1; and related clinical outcomes including respiratory function such as forced expiratory volume in one second (FEV1), forced vital capacity (FVC), asthma control and symptoms such as dyspnoea, coughing, wheezing and chest tightness.

Citations from literature databases were imported into referencing software Endnote X7.7 (Clarivate Analytic, Philadelphia, PA, USA). All studies retrieved by the search strategy were initially assessed for relevance to the review based on the title using inclusion and exclusion criteria. Articles considered not relevant based on title were coded NR (not retrieve) with the reason noted. Articles considered relevant, or unclear were coded R (retrieve). Further assessments of the retrieved articles were according to the abstract, keywords and MeSH terms, using the inclusion and exclusion criteria. Again, articles were coded as either NR with the reason or R. Retrieved full text articles were then assessed for inclusion criteria. If there was doubt as to whether an article met the defined inclusion criteria according to the title, abstract, keywords and MeSH term, the full article was assessed for clarification.

### 2.3. Study Quality

Eligible studies were assessed in terms of the methodological quality based on a standardised critical appraisal checklist designed by the American Dietetic Association [16]. The tool considered the reliability, validity, as well as generalisability of the included studies. No study was excluded due to poor quality. The two reviewers (BH and BB) then made final decisions on the included studies by cross-checking results. In cases of disagreement on the inclusion of a study, the other independent reviewers decided on the inclusion or exclusion of the study. Studies that were excluded at this stage were recorded with the reason noted.

### 2.4. Data Extraction and Study Synthesis

Study details were extracted and recorded into a custom-designed database. Data extracted included title, authors, country, study design, participant characteristics, study factor (e.g., dosage/dietary intake of fruits and vegetables), main outcome measures, findings including statistical significance, analysis with adjustment for confounding factors, and limitations.

### 2.5. Statistical Methods

A meta-analysis was used to evaluate the association between fruit and vegetable intake and risk of asthma and/or wheezing. Only studies that met the following inclusion criteria were included in the meta-analysis: (a) fruit and vegetable intake reported; (b) the odds ratio (OR) or the relative risks and the corresponding 95% confidence intervals (CI) were reported. However, due to the heterogeneity of study designs and differences in exposure and outcome assessments, meta-analysis of all of these studies were not possible. The analysis was performed for the total number of adults and children together, and pregnant women. To assess the risk of asthma and/or wheezing, the risk estimate from each study, weighted by the inverse of variance, was pooled. Appreciable heterogeneity was assumed if *I*^2^ > 50 and *p* < 0.1. Meta-analysis was performed using random effect modelling if *I*^2^ > 50 and fixed effect modelling was used if *I*^2^ < 50. Most studies assessed dietary intake with a validated food frequency questionnaire (FFQ), and other studies used a dietary habit questionnaire, food diaries or 24 h recall. Some studies used an FFQ with limited fruit and vegetable items such as Rosenlund et al. [17], while other studies used an FFQ which included over 50 items, such as Shaheen et al. [18] (>200 items), Romieu et al. [19] (108 items), and Protudjer et al. [20] (72 items). In addition, some of the FFQs were modified for use in children [21]. Since the included studies used different methods in reporting fruit and vegetable intake (i.e., >4 times/week vs. never, quartile 4 vs. quartile 1, daily intake vs. never, etc.), in order to include more studies in the meta-analysis, two terms were defined: high fruit and/or vegetable intake (the group that had the highest intake of fruit and vegetables in each study) vs. low fruit and/or vegetable intake (the group that had the lowest intake of fruit and vegetables in each study). Table 1, Table 2 and Table 3 show how the variables are contrasted in different studies.

## 3. Results

**Figure 2:**
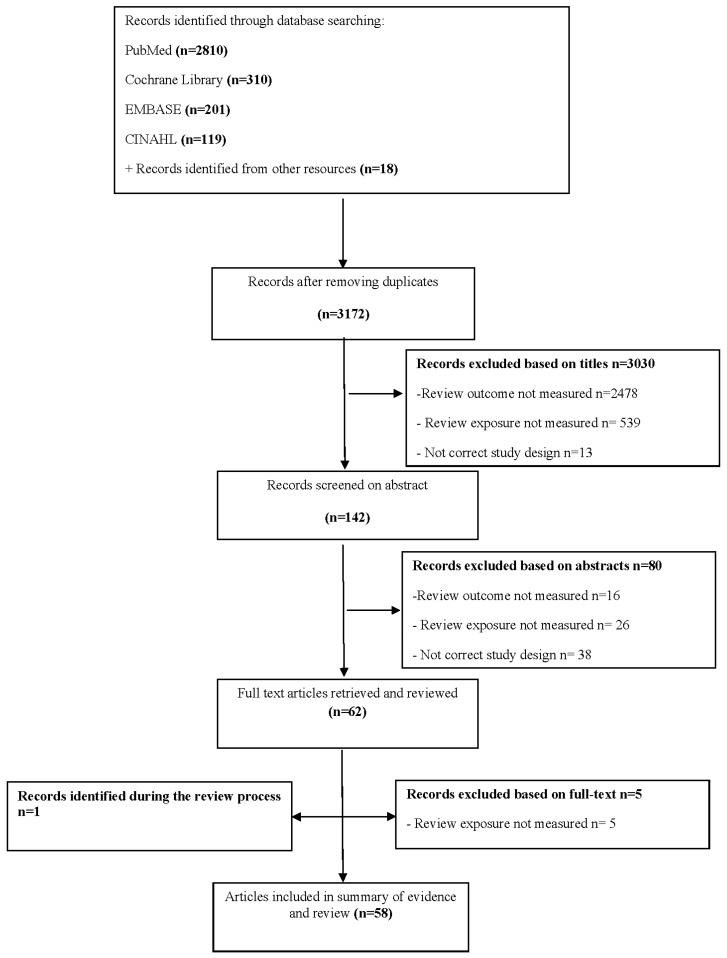
Preferred Reporting Items for Systematic Reviews and Meta-Analysis (PRISMA) flowchart of studies to include in systematic review of the association between fruit and vegetable intake and asthma.

Initially, 3194 abstracts were identified by the search strategy. After removing duplicates and screening the titles, 142 articles were retrieved for abstract review. Based on the abstracts, 80 articles were excluded based on outcomes (*n* = 16), exposures (*n* = 26) or study design (*n* = 38). After reviewing the full-texts, five articles were excluded as fruit and vegetable intakes were not reported, and also one study was additionally included during the review process. Finally, Fifty-eight articles were included in the review (Figure 2).

### 3.1. Characteristics of Included Studies

More than half of the studies were performed in children (*n* = 28), adolescents (*n* = 3) or both (*n* = 10), with only 17 conducted in adults. Cross-sectional design was most commonly used (Table 1) with 8 case-control studies (Table 2), 13 cohort studies (Table 3) and 7 clinical trials (Table 4). The majority of studies were conducted in UK (*n* = 8) [1,18,22,23,24,25,26,27], but also in Australia (*n* = 5) [28,29,30,31,32], Greece (*n* = 5) [13,33,34,35,36], Spain (*n* = 4) [37,38,39,40], Italy (*n* = 4) [9,41,42,43], Brazil (*n* = 3) [44,45,46], Netherlands (*n* = 3) [21,47,48], USA (*n* = 3) [9,49,50], Canada (*n* = 2) [20,51], Finland (*n* = 2) [52,53], India (*n* = 2) [54,55], Japan (*n* = 2) [56,57], Mexico (*n* = 2) [19,58] Sweden (*n* = 2) [17,59], Taiwan [60,61] (*n* = 2), Albania [62], China [63], Colombia [64], Germany [7], Ireland [65], Norway [66], Portugal [67], Saudi Arabia [68], and Singapore [69]. In addition, one study [70] used data from 20 countries. A total of 496,741 participants were included from cross-sectional studies, 2139 cases and 2739 controls from case-control studies, 105,789 individuals with the mean follow-up of 9.53 years from cohort studies. In total, 7109 participants were included in clinical trials with a mean intervention period of 141 days ranging from 3 to 365 days. The methodological quality of 41 studies was positive, and 16 studies were neutral.

### 3.2. Studies Conducted in Adults

Four cohort [22,52,58,59], two case-control [18,23], eight cross-sectional studies [31,32,42,55,57,62,67,69] and three experimental trials [28,29,30] assessed the association of fruit and vegetable intake and asthma or asthma-related symptoms in adults. Fruit and vegetable intake was reported to have beneficial associations with wheeze, or asthma in eight studies [18,22,28,29,42,55,59,62]; one study [69] found no significant relationship; and eight studies [23,30,31,32,52,57,58,67] reported mixed results. Most of the studies measured total fruit and vegetable intake; however, one cross-sectional study examined only vegetable intake [42], three cohort studies assessed only fresh fruit intake [22,59], and one study [52] analysed the consumption of orange, apple, grapefruit, onion, white cabbage, berries, and juices.

#### 3.2.1. Cohort Studies

In terms of prospective studies, two studies [22,59] reported fruit and vegetable intake was inversely associated with asthma. Likewise, Knekt et al. [52] found that higher dietary flavonoid intake (measured by intakes of orange, apple, grapefruit, onion, white cabbage, berries, juices) was associated with lower incidence of asthma. The strongest associations were noted for apple and orange intakes and asthma. Another study [58] reported inverse associations between intakes of tomato, carrots, leafy vegetables and asthma. No cohort studies in adults reported associations between fruit and vegetable intake and immune function in asthma.

#### 3.2.2. Case-Control Studies

Two case-control studies [18,23] reported that fruit and/or vegetables intake was inversely associated with asthma risk in adults. No case-control studies in adults reported associations between fruit and vegetable intake and immune function in asthma.

#### 3.2.3. Cross-Sectional Studies 

In line with these findings, a cross-sectional study by La vecchia et al. [42] reported that vegetable consumption was inversely associated with bronchial asthma. While Priftanji et al. [62] reported that fruit and vegetable intake between meals can have protective effects against possible allergic asthma. Another cross-sectional study [31] showed that apple and pear intake was inversely associated with current asthma, ever asthma, and BHR, and no significant association was observed regarding vegetable intake and asthma. In contrast, another cross-sectional study [69] failed to observe any significant association between fruit and vegetable intake and FEV_1_ and FVC. Barros et al. [67] found no significant associations between fruit and vegetable intake and exhaled nitric oxide (F_E_NO) in adults.

#### 3.2.4. Experimental Trial

We have previously investigated the effects on both lung function and airway inflammation following a LOA (low antioxidant) diet, which involved restriction of dietary fruit and vegetable intake [28]. FEV_1_ and FVC % predicted values decreased (*p* < 0.01) and sputum neutrophils % increased (*p* < 0.05) following the diet. The study also reported that treatment with tomato juice and tomato extract reduced airway neutrophils and sputum neutrophil elastase activity. Similarly, Baines et al. [29] showed that antioxidant withdrawal, via fruit and vegetable restriction, leads to upregulation of genes involved in the inflammatory and immune responses including the innate immune receptors TLR2, IL1R2, CD93, ANTXR2, and the innate immune signalling molecules IRAK2, 3, MAP3K8 and neutrophil proteases. In another trial [30], we found that subjects on a LAO diet (involving fruit and vegetable restriction) were 2.26 times as likely to have an asthma exacerbation at any time in comparison with the high antioxidant diet group. Lung function also decreased following antioxidant withdrawal in this study. There were no improvements in airway and systemic inflammation, lung function and asthma control after tomato extract supplementation in this study.

### 3.3. Studies Conducted in Children and Adolescents

Forty-one studies including nine cohort [21,33,37,41,47,48,53,56,65], six case-control [19,20,44,45,49,68], 22 cross-sectional studies [1,7,9,13,17,26,27,34,35,36,39,40,43,46,50,51,54,61,63,64,66,70], and four experimental trials [24,25,38,60] assessed the effects of fruit and vegetable intake on asthma or asthma-related symptoms in children and adolescents. An inverse association of fruit and vegetable intake and asthma or wheeze was reported in 22 studies [1,9,13,19,21,26,33,35,38,39,41,43,45,49,50,54,60,63,65,66,70,71], while seven studies [7,24,27,34,36,53,56] did not observe any significant association. Twelve studies [17,20,37,40,44,46,47,48,51,61,64,68] found mixed results. Four studies [9,19,25,49] reported on immune responses to fruit and vegetable intake in children in relation to asthma, with all studies showing a protective effect on systemic or airway inflammation. The majority of studies analysed total fruit and vegetable intake, though five cross-sectional studies assessed the consumption of fruit only [1,27,43,64] or citrus fruit plus vegetables [7]. Additionally, one cohort study [41] assessed the intake of cooked vegetables, salads, tomatoes, fresh fruit, citrus fruit and kiwi, and one birth cohort study [53] measured food-based antioxidant intake.

#### 3.3.1. Cohort Studies

In a one-year prospective study [41], intake of tomatoes and all fruits and citrus fruit alone had a protective effect on shortness of breath. Similarly, a cohort study [33] that followed children from birth up to 18 years of age reported that daily consumption of fruit and vegetables over the last 12 months was inversely associated with current asthma at 18 years. Another birth cohort study [48] showed that intakes of fresh fruit were inversely associated with asthma symptoms, while, no significant association was observed between cooked vegetable intake and asthma symptoms. No cohort studies in children reported associations between fruit and vegetable intake and immune function in asthma

Four cohort studies addressed the association between maternal fruit and vegetable intake during pregnancy and risk of asthma-related outcomes in their children. One study [65] reported an inverse association between asthma incidence in children and maternal fruit and vegetable intake in pregnancy. Another study [21] reported that maternal apple intake had protective effects on ever wheeze, ever asthma, and doctor-confirmed asthma in the children; however, no consistent associations were observed between childhood outcomes and maternal vegetable consumption. In contrast, a study conducted by Chatzi et al. [37] showed that consumption of vegetables more than eight times per week was inversely correlated with persistent wheeze, while, no association was found regarding fruit intake and wheeze. Two studies [53,56] found no significant association regarding maternal fruit or vegetable intake and risk of wheeze in the offspring. Willers et al. [47] demonstrated that fruit intake had a borderline significant association with wheeze. This study also reported that vegetable intake was positively associated with asthma symptoms in children. 

#### 3.3.2. Case-Control Studies

In terms of case-control studies, four studies [20,44,49,68] reported that consumption of vegetables was negatively associated with odds of asthma; however, no difference was observed regarding fruit intake among the groups. In contrast, one study [45] found that regular consumption of fruit in the last month was associated with lower risk of having persistent asthma, while there was no difference in vegetable consumption between the two groups. A follow-up case-control study by Romieu et al. [19] reported that the fruit and vegetable index (FVI) was positively related to FEV_1_ and FVC. A 1-point increase in FVI was associated with a 105 mL (nearly 5%) increase in FVC. For each one-unit increase in FVI there was a significant decrease in IL-8 levels in nasal lavage. Similarly, a recent study [49] reported that increased vegetable consumption is negatively associated with serum IL-17F.

#### 3.3.3. Cross-Sectional Studies

The majority of cross-sectional studies reported beneficial associations of fruit and vegetable intake with lung function (FEV_1_ and FVC) [26,50], wheeze [13,35,43,54] and asthma [1,9,39,63,66] as well as with F_E_NO levels [9] as a marker of eosinophilic airway inflammation. However, some studies did not observe any association between fruit and vegetable intake and asthma in children [7,17,27,36]. Several cross-sectional studies found an inverse association between vegetable consumption and asthma-related symptoms, although, they did not observe any significant association regarding fruit intake and asthma [37,40,51,61]. In contrast, a recent study [46] reported fruit intake was inversely related to odds of asthma, while, no association was found between vegetable intake and asthma. Garcia et al. [64] found a negative association between fruit intake and asthma in adolescents. An international study of 20 countries [70] reported consumption of cooked green vegetables and raw green vegetables was significantly associated with fewer wheezers in non-affluent countries, and fruit intake was associated with a low prevalence of current wheeze in affluent and non-affluent countries.

#### 3.3.4. Experimental Studies

Lee et al. [60] described a 16-week trial of 192 children with asthma who received fruit and vegetable capsules + fish oil + probiotic vs. placebo and reported the supplement group had a significantly higher increase in FEV_1_, FVC and FEV_1_:FVC ratio compared to placebo. The proportion of children using inhaled glucocorticoids decreased following the supplementation, though increased in the placebo group. In another trial by Garcia et al. [25] 32 asthmatic children were randomly allocated to one of four groups: having an apple or a banana or an apple + banana in addition to their normal diet, or the control group (usual diet). The study reported 18% lower FeNO levels in Groups 2 (adding banana) and 3 (adding banana + apple) following the intervention. In a study by Calatayud et al. [38], 104 children aged 1–5 years with current asthma participated in a nutritional education programme based on the traditional Mediterranean diet for one year. The authors reported that fruit and vegetable intake increased significantly with a concomitant decrease in inhaled glucocorticoids use. In contrast, a one-year trial by Fogarty et al. [24] conducted in asthmatic children found that there was no difference in the prevalence of wheezing, exercise-induced wheeze, or nocturnal cough between children who were instructed to add an extra piece of fruit to their diet compared to the control group. 

### 3.4. Findings from Meta-Analysis

**Figure 3:**
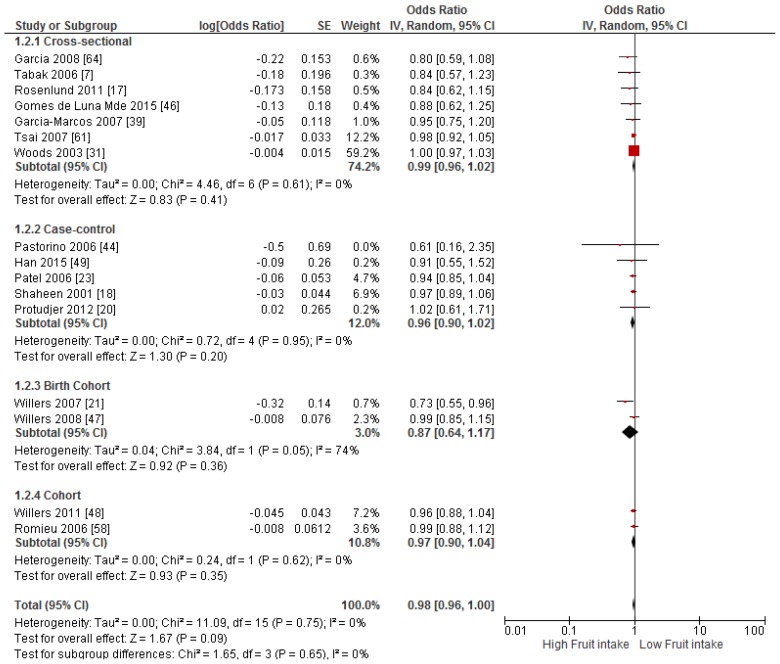
Meta-analysis of the association between fruit intake and risk of prevalent asthma.

**Figure 4:**
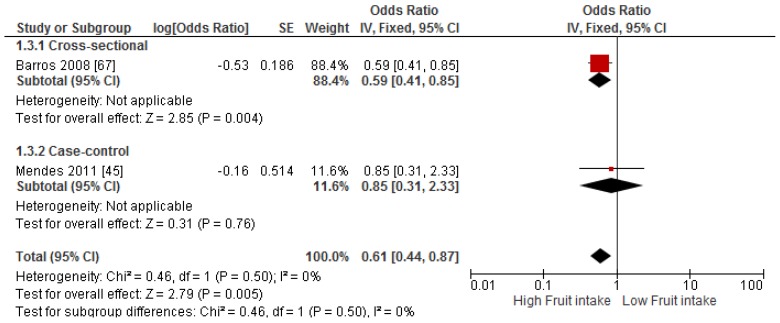
Meta-analysis of the association between fruit intake and severity of asthma.

**Figure 5:**
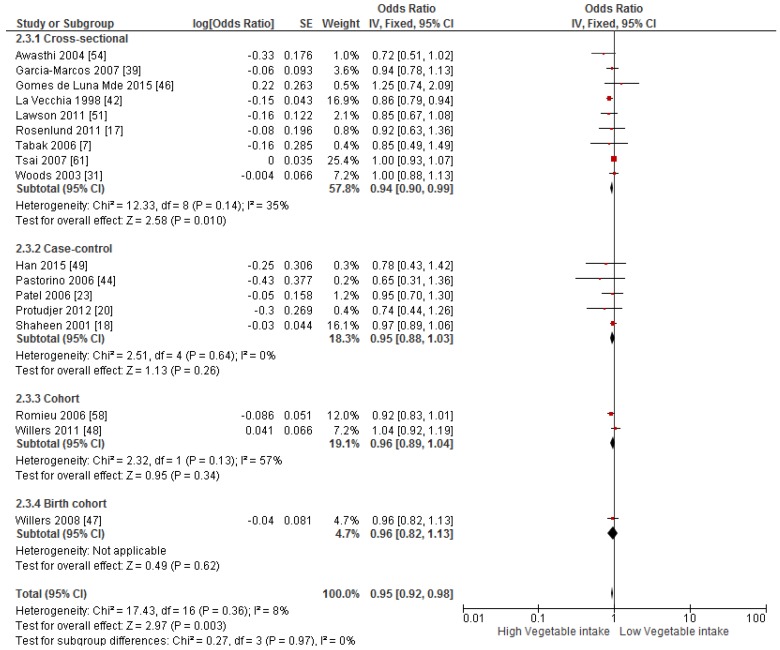
Meta-analysis of the association between vegetable intake and risk of prevalent asthma.

**Figure 6:**
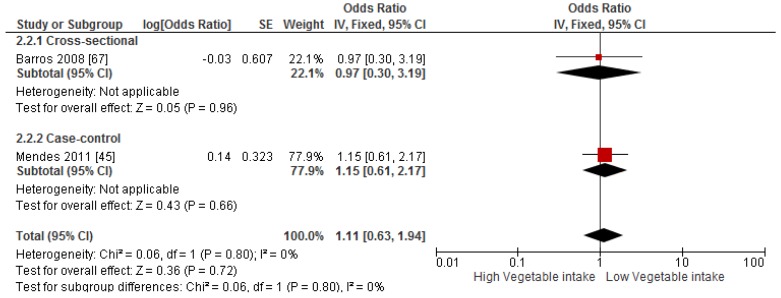
Meta-analysis of the association between vegetable intake and severity of asthma.

**Figure 7:**
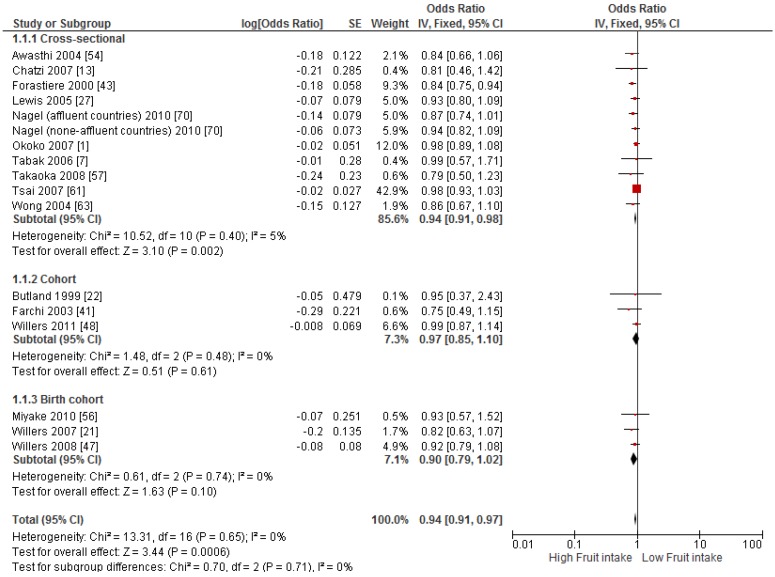
Meta-analysis of the association between fruit intake and risk of prevalent wheeze.

**Figure 8:**
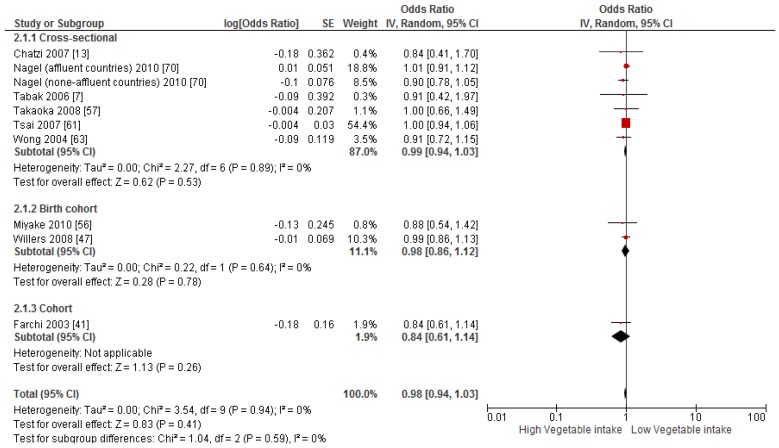
Meta-analysis of the association between vegetable intake and risk of prevalent wheeze.

**Figure 9:**
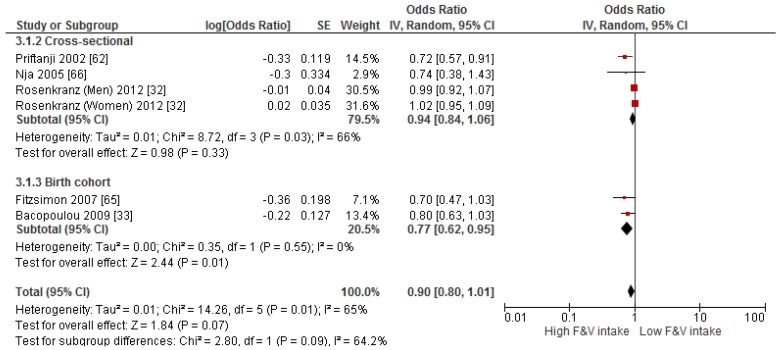
Meta-analysis of the association between fruit and vegetable intake and risk of prevalent asthma.

Primary prevention studies that reported the OR associated with the risk of prevalent asthma/wheeze were analysed separately to secondary prevention studies that reported the OR associated with asthma severity (*n* = 2) or wheeze severity (*n* = 1). Meta-analyses of 17 primary prevention studies revealed no significant association between fruit intake and risk of prevalent asthma (OR = 0.98; 95% CI: 0.96–1.0, *p* = 0.09, *I*^2^ = 8) (Figure 3). However, intake of fruit was inversely associated with the severity of asthma in secondary prevention studies [45,67] (OR = 0.61; 95% CI: 0.44–0.87, *p* = 0.005, *I*^2^ = 0) (Figure 4). Vegetable intake was negatively related to the prevalence of asthma (OR = 0.95; 95% CI: 0.92–0.98, *p* = 0.003, *I*^2^ = 8) (Figure 5), while it was not related to the severity of asthma in secondary prevention studies [45,67] (OR = 1.11; 95% CI: 0.63–1.94, *p* = 0.72, *I*^2^ = 0) (Figure 6). Fruit intake was also negatively associated with risk of prevalent wheeze (OR = 0.94; 95% CI: 0.91–0.97, *p* < 0.0001, *I*^2^ = 0%) (Figure 7). However, no significant relationship was found between vegetable intake and risk of prevalent wheeze (OR = 0.98; 95% CI: 0.94–1.03, *p* = 0.41, *I*^2^ = 0%) (Figure 8). Meta-analysis of the association between total fruit and total vegetable intake with the severity of wheeze was not possible as there was only one secondary prevention study [37]. A meta-analysis of six primary prevention studies that reported fruit and vegetable intake together showed no significant relationship with the risk of prevalent asthma (OR = 0.90; 95% CI: 0.80–1.01, *p* = 0.07, *I*^2^ = 65%) (Figure 9). No meta-analysis was possible for immune markers as well as respiratory infection, due to the lack of available studies. Moreover, meta-analysis on the association between fruit and/or vegetable intake and respiratory function was not possible, as studies used heterogeneous methods in reporting these outcomes.

## 4. Discussion

This systematic review and meta-analysis is the first aimed at investigating the effects of fruit and vegetable consumption on risk of asthma and wheezing and immune responses (including immune responses to virus infection and inflammation) in asthma and wheezing. We found that the majority of studies (*n* = 8 in adults and *n* = 22 in children) reported a protective effect of a high fruit and vegetable diet on asthma and/or wheeze. Twenty studies (*n* = 8 in adults and *n* = 12 in children) reported mixed results, as they found a negative association between intake of fruit only or vegetable only and risk of asthma and/or wheeze. Eight studies (*n* = 1 in adults and *n* = 7 in children) failed to show any beneficial effects of fruit and vegetable intakes on risk of asthma and wheeze. In the meta-analysis, fruit intake was not associated with the risk of prevalent asthma (*p* > 0.05); however, a negative relationship was observed between consumption of fruit and asthma severity. Intake of vegetables was inversely associated with the prevalence of asthma, while there was no association in secondary prevention studies. In addition, fruit intake was negatively associated with the risk of prevalent wheeze, while no significant relationship was found between vegetable consumption and wheeze prevalence. Fruit and vegetable intake together showed no significant relationship with the risk of prevalent asthma. Meta-analyses of immune response parameters and respiratory infections were not possible due to lack of studies reporting on these markers. 

Several mechanisms for the protective effects of fruit and vegetables on asthma and lung function have been suggested. Fresh fruit and vegetables are rich dietary sources of antioxidants such as vitamin C, E and β-carotene as well as flavonoids, isoflavonoids and polyphenolic compounds [1,7]. It has been reported that oxidative stress is elevated in asthma and increases further during acute asthma exacerbations [72,73,74], so a high intake of antioxidants may be beneficial. Vitamin C is a major antioxidant in the extracellular respiratory lining fluid that protects immune cells from oxidative stress, and may also contribute to lung growth and development and reduce airway hyper-reactivity, both of which are determinants of childhood and adult lung function [50]. The potential for vitamin C to prevent asthma-related outcomes is illustrated by a cross-sectional study that documents the protective effects of dietary vitamin C against wheezing and shortness of breath [43]. Vitamin E can be found in various fruits and vegetables, including corn, tomato, spinach, broccoli, kiwifruit, and mango [75]. One study reported that consumption of vitamin E was negatively correlated with forced expiratory flow, which is a measure of small airway flow [50]. Another group of low molecular weight antioxidants found in fruits and vegetables are carotenoids. Lycopene, present in high concentrations in tomatoes, red fruits, watermelons, apricots and pink grapefruit, is the most potent antioxidant among the carotenoids [76]. It has been suggested that oral intakes of lycopene reduce both oxidative stress and the pathophysiological features of asthma such as airway smooth muscle contraction, induction of BHR, and mucus hypersecretion [76]. Flavonoids are a group of polyphenols found in fruits and vegetables that have potent antioxidant as well as anti-inflammatory effects. One study showed that the incidence of asthma was lower at higher total intakes of flavonoids [52]. Moreover, it has been reported that a specific type of flavonoid, called “khellin”, was used traditionally in asthma treatment because of its bronchodilator activity [52]. It is likely that the positive effects of fruit and vegetables can be attributed to the combination of these nutrients, which are present in high concentrations in this natural food source. Moreover, evidence indicates that paediatric asthma, which is allergic in 80% of cases [77], may benefit from fruit and vegetable intake, as several studies reported lower rates of wheezing and allergic rhinitis in children who consumed antioxidant-rich foods daily [36,48,78]. Similarly, high antioxidant intake is related to enhanced pulmonary function and reduced chronic respiratory symptoms in children, especially those exposed to high amounts of air or smoke pollution [36]. Studies also suggest that antioxidants might affect immune function and allergic reactions [36,78]. A study of school-aged children [79] reported that asthma and allergic rhinitis were inversely correlated with serum levels of antioxidants compared to healthy children. Similar results were also observed in adults [80].

This systematic review has highlighted the lack of available data regarding the effects of fruit and vegetable intake on immune responses in asthma. Asthma is a chronic inflammatory disease, involving activation of a variety of immune cell types and increased oxidative stress. Oxidative stress occurs in asthma due to the excessive release of free radicals from activated inflammatory cells and is regarded as one of the critical factors involved in the chronic inflammatory process in both the airways and in the systemic circulation of asthmatics [9,11,74]. Viral respiratory infections are a key contributor to inflammation and oxidative stress in asthma and it has been shown that innate immune responses are impaired in asthmatic adults and children [81], therefore, patients with asthma are more vulnerable to virus infections compared with non-asthmatic controls [81,82]. Consequently, food such as fruits and vegetables, which have the potential to reduce inflammation and oxidative stress due to their anti-inflammatory and anti-oxidative properties, may be beneficial in asthma. Data from three observational studies [9,19,49] in children examined the association between fruit and vegetable intake and inflammation. In each case, there was an association between airway and/or systematic inflammation and intake of fruit and/or vegetable. A cross-sectional study by Cardinale et al. [9] reported that salad intake was negatively associated with F_E_NO levels. Similarly, an inverse association between fruit and vegetable intake and IL-8 levels in nasal lavage [19], and between vegetable intake and serum IL-17F [49] was reported by two case-control studies. Only one intervention study in children [25] has examined effects of fruit on inflammation, and reported that having a banana or a banana and apple for one month resulted in a statistically significant 18% reduction in F_E_NO levels. However, fruit and vegetable intake was not related to F_E_NO levels in adults [67]. In addition, two intervention studies have examined the effect of withdrawal of antioxidant-rich foods (in particular fruit and vegetables) from the diet, on immune responses in adults with asthma [28,30]. In one study, antioxidant withdrawal resulted in increased airway neutrophils [28] and upregulation of inflammatory and immune response genes in sputum cells, including the innate immune receptors TLR2, IL1R2, CD93, ANTXR2, the innate immune signalling molecules IRAK2, IRAK3, MAP3K8 and neutrophil proteases MMP25 and CPD [29]. The major dietary change in these studies was a decrease in fruit and vegetable consumption to a level, which is representative of the typical western diet, which is alarming considering the negative consequences in the airways [28]. 

There are several additional points that warrant consideration. Firstly, several proteins from fruits and vegetables are similar to pollen allergens and may play a critical role in the pollen–fruit/vegetable cross-reactivity. For instance, some allergens in apples, pears, various stone fruits, carrots, and peanuts are homologous to the major birch pollen allergen, and can cause allergic symptoms when consumed [17]. Therefore, children are less likely to eat them if they cause immediate symptoms. Such disease-related modification of diet may affect the observed associations between intake of certain fruits or vegetables and asthma [17]. In one study [17], 45% of asthmatic children had sensitization to birch pollen, and the authors reported that all negative associations between fruit and vegetable intake and asthma were no longer significant when children with food-related allergic symptoms were excluded; Secondly, it should be noted that fruit and vegetable consumption is associated with an overall healthier lifestyle. In particular, it has been reported that people with higher fruit and vegetable intakes are more likely to be non-smokers or ex-smokers [83], or perform more physical activity [84]. However, most of the included studies were adjusted for important lifestyle factors, such as smoking and/or physical activity status (see Supplementary Materials). 

The present systematic review has limitations that should be considered. Primarily, the criteria used for asthma diagnosis were inconsistent. Skin-prick testing and different questionnaires were used in some studies, while in other studies, asthma was diagnosed by a physician. Dietary assessment methods also varied among the studies. Some studies used a FFQ, whereas, the other studies used 7-day food records, 24 h recalls or other questionnaires. Various methods were also used to define and compare high and low intakes of fruit and vegetables (i.e., daily versus never, >3 times/week versus <3 times/week, lowest versus highest tertile of intake, >3 times/day versus <2 times/day), and thus dose-response relationships could not be assessed. Estimation of diet was also varied across the included studies, as some studies reported individual foods, while a few studies reported the dietary pattern, such as Mediterranean diet. However, only studies that reported data on fruit and vegetable intake were included. Dissimilar populations (i.e., children, adolescents, young adults and elderly) were also observed among the studies, and more than half of the studies were performed in children. The results have been presented according to study population and age, and birth cohort studies were assessed separately in the meta-analysis; however, due to the limited number of studies available for inclusion in the meta-analyses, separating the analyses for adults and children was not possible. Moreover, unmatched categories of diet exposure were observed; for example, some studies addressed the effects of total fruit and vegetable consumption [26,31,50,62], while a few studies [1,27,43,64] investigated the association between intake of a specific type of fruit and/or vegetable and asthma. Some studies also reported only total fruit intake or total vegetable intake. Therefore, only studies that reported total fruit and/or total vegetable intake were included and assessed in the meta-analysis. Moreover, some studies investigated diet as a risk factor for asthma (primary prevention), while other studies investigated diet as a disease modifier (secondary prevention). However, these different study types were assessed separately in the meta-analyses. In addition, adjustment for confounders in individual studies was performed using different covariates (see Supplementary Materials). For example, gender and physical activity were adjusted in some studies, and not adjusted for in other studies. As such, it is not surprising that the protective effect of fruit and vegetables was not reported by all included studies [7,17,24,34,53,56]. Furthermore, one cohort [48] and one cross-sectional study [61] demonstrated an increased risk of asthma as vegetable or fruit consumption increased. Nonetheless, there were enough studies to see a significant protective effect of fruit and vegetables against asthma and/or wheeze using meta-analysis. Finally, we cannot rule out the possible influences of potential factors such as genetics, race and ethnicity on the association between fruit and vegetable intake and asthma. Moreover, since most of the included studies had cross-sectional design, the results could be influenced by reverse causality bias. The main strength of this systematic review is the extensive systematic literature search, clear inclusion criteria and an explicit approach to collecting data, thorough examination of the evidence, inclusion of both adults and children, inclusion of studies with various designs and meta-analysis.

## 5. Conclusions

In summary, overall, the findings suggest that high intakes of fruit and vegetables may have beneficial effects in asthma. However, some studies failed to attain similar results. Further studies with cohort design that differ in regards to genetic susceptibility and ethnicity/race as well as well-designed intervention trials are warranted to accurately address the effects of fruit and vegetable consumption on risk of asthma development and their role in managing asthma. More evidence is also needed from laboratory studies to identify the biological mechanisms responsible for the effects of fruit and vegetable intake on the development and management of asthma.

## Figures and Tables

**Table 1:** Summary of cross-sectional studies on the association between fruit and vegetable intake and asthma.

Author (Year)	Food Measured	Study Population	Age Group (Year)	Tool for Asthma Diagnosis	Dietary Assessment Methods	Variables Contrasted	Outcomes
Cook et al. [26], 1997	F & V	2650	8–11	Questionnaire	FFQ ^a^	>1 time/day vs. never	↑ fresh fruit, salad, green vegetables consumption: ↑ FEV1, ↔ wheeze
La vecchia et al. [42], 1998	Vegetables	46,693	≥15	Questionnaire	FFQ	Highest (>7 serving/week) vs. lowest (<7 serving/week) tertiles	↑ vegetable consumption: ↓ bronchial asthma
Forastiere et al. [43], 2000	Fruits	4104	6–7	ISAAC questionnaire	Questionnaire on dietary habits, citrus fruit consumption	5–7 times/week vs. <1 time/week	↑ fruit: ↓ any wheeze, ↓ shortness of breath with wheeze
Priftanji et al. [62], 2002	F & V	2653	20–44	Questionnaire,	Questionnaire on dietary habits	At least once a week	↑ taking fruit and vegetables: ↓ possible allergic asthma
Gilliland et al. [50], 2003	F & V	2566	11–19	Pulmonary function testing	FFQ	≤lowest vs. highest intake decile	↓ intakes of all fruit juices: ↓ FEV1 and FVC among boys, ↓ intake of vegetable: ↓ FVC in girls, ↔ other respiratory symptoms
Woods et al. [31], 2003	F & V	1601	20–44	ECRHS questionnaire	FFQ	1–2 piece of apples, pears and berries/day and 2–4 servings leafy green vegetables and tomatoes/day	↑ consumption of apples and pears: ↓ current asthma vegetable intake: ↔
Awasthi et al. [54], 2004	F & V	3000	6–7 and 13–14	ISAAC questionnaire	Validated questionnaire	F: ≥3 times/day V: ≥1 time/week	↑ intakes of vegetables and fruits: ↓ wheeze
Wong et al. [63], 2004	F & V	10,902	10	Questionnaire, skin-prick test	Questionnaire on F & V intakes	F: more than once daily vs. <once daily; V:more than once a week vs. <1 per week	↑ intakes of fruit and vegetables: ↓ wheeze
Lewis et al. [27], 2005	Fruits	11,562	4–6	Questionnaire	Questionnaire	≥21 portions/week vs. 0 portions/week	Fruits: ↔ wheeze
Nja et al. [66], 2005	F & V	502	6–16	Questionnaire, skin-prick test	Questionnaire	Daily intake vs. occasionally	↑ intakes of fruit and vegetables: ↓ asthma
Tabak et al. [7], 2006	Citrus and V	598	8–13	ISAAC questionnaire	FFQ	F: Highest (287 g/day vs. lowest (79 g/day) tertiles; V: Highest (140 g/day vs. lowest (53 g/day) tertiles	citrus fruits, vegetables: ↔
Cardinale et al. [9], 2007	F & V	130	6–7	Doctor-diagnosed asthma	FFQ	Always vs. never	↑ intakes of fruit: ↓ asthma ↑ intakes of salads: ↓ F_E_NO
Chatzi et al. [13], 2007	F & V	690	7–18	ISAAC questionnaire, family history of allergic disease	FFQ	>1 time/day vs. <1 time/day	↑ intake of grapes, oranges, apples and fresh tomatoes: ↓ wheezing
Garcia-Marcos et al. [39], 2007	F & V	20,106	6–7	ISAAC questionnaire	Questionnaire	≥3 times/week vs. never	↑ intakes of fruit and vegetables: ↓ COA, ↓ CSA
Okoko et al. [1], 2007	Fruits	2640	5–10	ISAAC questionnaire	FFQ	>1 serving/day vs. <1 serving/month	↑ intakes of apples: ↓ ever-asthma ↑ intake of bananas and apples: ↓ ever wheeze and current wheeze
Tsai et al. [61], 2007	F & V	2218	11–12	ATS questionnaire	FFQ	Daily intake vs. never	↑ Fruit intakes: ↓ wheezing without cold, ↓ asthma, ↑ vegetable consumption: ↑ asthma
Barros et al. [67], 2008	F & V	174	>16	Doctor-diagnosed asthma and questionnaire	FFQ	F: <178.4 g/day vs. >304.97 g/day; V: <211.54 g/day vs. >426.63 g/day	↑ consumption of fresh fruit: ↓ non-controlled asthma; vegetable intake: ↔; F&V: ↔ exhaled NO
Castro-Rodriguez et al. [40], 2008	F & V	1784	4.08	Questionnaire	FFQ	>3 times/week vs. never	↑ intake of vegetable: ↓ wheezing; fruits intake: ↔
Garcia et al. [64], 2008	Fruits	3256 children and 3829 adolescents	6–7 and 13–14	ISAAC questionnaire	Questionnaire on dietary habits	≥3 times/week vs. occasionally	↑ fruit consumption: ↓ current asthma symptoms among the 13–14 year age-group
Takaoka et al. [57], 2008	F & V	153 females	Mean 21	Doctor -diagnosed asthma, ISSAC/ECRHS questionnaire	FFQ	almost daily vs. never	↑ intake of fruit: ↓ wheeze vegetables: ↔
Nagel et al. [70], 2010	F & V	50,004 ^b^	8–12	ISAAC questionnaire	FFQ	≥3 times/week vs. never/occasionally	↑ consumption of green vegetables: ↓ wheezers in non-affluent countries only; ↑ fruit intake: ↓ prevalence of current wheeze in affluent and non-affluent countries
Arvaniti et al. [34], 2011	F & V	700	10–12	ISAAC questionnaire	FFQ	At least once/day	Fruits and vegetables: ↔ asthma
Lawson et al. [51], 2011	F & V	4726	11–15	Doctor-diagnosed asthma	Validated questionnaire	High vs. low consumption	↑ vegetable consumption: ↓ current asthma; fruit intake: ↔
Rosenlund et al. [17], 2011	F & V	2447	8	Doctor-diagnosed asthma	FFQ	Quartile 4 (7.1 serving/day) vs. quartile 1 (1.8 serving/day)	Fruits and vegetables: ↔; apples/pears, carrots: ↓ asthma
Rosenkranz et al. [32], 2012	F & V	156,035	≥45	Questionnaire, self-reported information	FFQ	Quintile 5 vs. 1	↑ fruit and vegetable intake: ↓ asthma in men
Agrawal et al. [55], 2013	F & V	156,316	20–49	Questionnaire	FFQ	Daily intake vs. occasionally/never	↑ fruit and vegetable intake: ↓ asthma
Ng et al. [69], 2013	F & V	2478	≥55	Spirometry	SQFFQ	Once/day	Fruits and vegetables: ↔ respiratory function
Alphantonogeorgos et al. [35], 2014	F & V	1125	10–12	ISAAC questionnaire	KIDMED FFQ	Once/day	↑ intake of one fruit or fruit juice and vegetable: ↓ ever wheezing and current wheezing
Papadopoulou et al. [36], 2014	F & V	2023	9–10	Doctor-diagnosed asthma	SQFFQ	Daily vs. never	Fruits, vegetables: ↔ asthma
Gomes de Luna Mde et al. [46], 2015	F & V	3015	13–14	ISAAC questionnaire	Questionnaire	≥3 times/week vs. < times/week	↑ Fruit intake: ↓ asthma; vegetable intakes: ↔

^a^ Abbreviation: BMI, body mass index; COA, current occasional asthma; CSA, current severe asthma; ECRHS, the European community respiratory health survey screening questionnaire; F_E_NO, fractional exhaled nitric oxide; FFQ, food frequency questionnaire, F & V, fruits and vegetables; SQFFQ, semi-quantitative food frequency questionnaire; ISAAC, international study of asthma and allergies in childhood questionnaire; ^b^ 29 centres in 20 countries (ISAAC Phase II).

**Table 2:** Summary of case-control studies on the association between fruit and vegetable intake and asthma.

Author (Year)	Food Measured	Study Population	Age (Year)	Tool for Asthma Diagnosis	Dietary Assessment Methods	Variables Contrasted	Outcomes
Hijazi et al. [68], 2000	F & V ^a^	114 cases with a history of asthma and wheeze in the last 12 months and 202 controls	12	ISAAC questionnaire and skin test	SQFFQ	>3 time/day vs. <2	↓ vegetables intake: ↑ asthma; fruit intake ↔
Shaheen et al. [18], 2001	F & V	607 cases and 864 controls	16–50	Questionnaire	FFQ	≥5 times/week vs. <once/month	↓ Total fruit and total vegetable consumption: ↑ asthma
Patel et al. [23], 2006	F & V	515 cases and 515 controls	45–75	Physician diagnosed asthma	7-day food diaries	Consumption above the median (F: 132.1 g, V: 96.9 g vs. no consumption)	↑ intake of citrus fruit and total fruit intakes: ↓ asthma; vegetable intake ↔
Pastorino et al. [44], 2006	F & V	528	13–14	ISAAC questionnaire	Questionnaire about dietary habits	Weekly or daily vs. never consumption	↑ intake of cooked vegetables: ↓ asthma; fruit intake ↔
Romieu et al. [19], 2009	F & V	158 cases and 50 controls followed for 22 weeks	6–14	Physician diagnosed asthma	FFQ	F & V index = 0 vs. F & V index =4	↑ FVI: ↑ FEV1 and FVC; ↓ IL-8 in nasal lavage, ↓ FeNO level
Mendes et al. [45], 2011	F & V	104 cases with persistent asthma and 67 controls with intermittent asthma	2–12	Questionnaire	Dietary data collected during the last 30 days	Regular vs. occasional consumption	↑ consumption of fruits: ↓ persistent asthma
Protudjer et al. [20], 2012	F & V	149 cases and 327 controls from a Cohort study	8–10 and 11–14	Skin-prick test ≥3 mm and asthma symptoms	FFQ	High vs. low score (>6 times/day vs. Almost never)	↑ vegetable intake: ↓ allergic asthma, ↓ moderate/severe AHR; fruit intake: ↔
Han et al. [49], 2015	F & V	351 cases and 327 controls	6–14	Physician-diagnosed asthma and ≥1 episode of wheeze in the previous year	Questionnaire	Quartile 4 vs. quartile 1	↑ consumption of vegetables: ↓ asthma, ↓ serum IL-17F

^a^ Abbreviation: BMI, body mass index; FFQ, food frequency questionnaire, F & V, fruits and vegetables; ISAAC, international study of asthma and allergies in childhood questionnaire; MDS, Mediterranean diet score; SQFFQ, semi-quantitative food frequency questionnaire.

**Table 3:** Summary of cohort studies on the association between fruit and vegetable intake and asthma.

Author (Year)	Food Measured	Study Population	Age Group (Year)	Follow-Up (Year)	Tool for Asthma Diagnosis	Dietary Assessment Methods	Variables Contrasted	Outcomes
Butland et al. [22], 1999	Fresh fruit	11,352	0–33	33	Wheezing/whistling in the chest in the past doctor diagnosis	Validated questionnaire	>1 time/day vs. never	↑ Fresh fruit, salads or raw vegetables consumption: ↓ the frequent wheezing
Knekt et al. [52], 2002	Orange, apple, grapefruit, onion, white cabbage, berries, juices	382	30–69	20	Questionnaire	Dietary history	Quartile 4 vs. 1	↑ apple and orange intakes: ↓ asthma
Farchi et al. [41], 2003	Cooked vegetables, salads, tomatoes, fresh fruit, citrus fruit, kiwi	4104	6–7	1	ISAAC questionnaire	FFQ	>4 times per week vs. never	↑ Consumption of tomatoes, fruits and citrus fruit: ↓ shortness of breath
Romieu et al. [58], 2006	F & V	68,535 women	40–65	3	Questionnaire	FFQ	Quartile 4 vs. Quartile 1 (fruits: >336 vs. ≤145.3 g/day and vegetables: >90 vs. ≤39.3 g/day)	↑ Consumption of tomatoes, carrots, and leafy vegetables: ↓ asthma
Fitzsimon et al. [65], 2007	F & V	631 mother-child pair	3	3	Doctor-diagnosed asthma	SQFFQ	Quartile 4 (8.9 serving/day) vs. quartile 1(2.3 serving/day)	↑ Quartile of F & V intake in pregnancy: ↓ asthma in children
Willers et al. [21], 2007	F & V	1212 mother-child pair	At birth	5	ISAAC questionnaire	FFQ	>4 times/week vs. 0 ↔ 1 time/week	↑ Maternal apple intake: ↓ ever wheeze, ↓ ever asthma and doctor-confirmed asthma vegetables: ↔
Chatzi et al. [37], 2008	F & V	507 mothers and 468 children	6.5	6.5	Questionnaire on wheeze, whistling and skin-prick test	FFQ	Daily or weekly consumption vs. never	↑ Consumption of vegetables: ↓ persistent wheeze, fruits: ↔
Willers et al. [47], 2008	F & V	2832 mother-child pairs	3 month–8 year	8	ISAAC questionnaire	Questionnaire about both mother’s and child’s diet	Daily vs. rare intake	↑ Fruit intake: ↓ wheeze vegetables: ↔; F & V intake: ↔
Bacopoulou et al. [33], 2009	F & V	2133 children	From birth	18	Doctor-diagnosed asthma and questionnaire about detailed information on asthma	Validated questionnaire	Daily intake vs. never	↑ Fruit and vegetable intake: ↓ current asthma at 18 years
Miyake et al. [56], 2010	F & V	763 mother-child pair	16–24 month	2	ISAAC questionnaire	DHQ	F: Quartile 4 (290.8 g/day) vs. 1 (49.6 g/day) V: Quartile 4 (288.4 g/day) vs. 1 (90.9 g/day)	F & V intake: ↔ wheeze
Uddenfeldt et al. [59], 2010	Fruit	8066 females and males	16, 30–39, 60–69	13	questionnaire	Questionnaire about frequency of current consumption	Daily intake vs. never	↑ Fruit intake: ↓ asthma incidence
Nwaru et al. [53], 2011	Food-based antioxidants	2441 mother-child pair	5	5	ISAAC questionnaire	FFQ	Quantity of intake in diet	Food-based antioxidants: ↔ asthma
Willers et al. [48], 2011	F & V	4146 children	2–3 and 7–8	8	ISAAC questionnaire	Annual FFQ	Once weekly vs. long-term intake from age 2–8 years	↑ Fruit intake: ↓ asthma symptoms; cooked ↑ vegetables intake: ↑ asthma

^a^ Abbreviation: BMI, body mass index; DHQ, dietary habit questionnaire; FFQ, food frequency questionnaire, F & V, fruits and vegetables; ISAAC, international study of asthma and allergies in childhood questionnaire, SQFFQ, semi quantitative food frequency questionnaire.

**Table 4:** Summary of experimental studies on the association between fruit and vegetable intakes and asthma.

Author (Year)	Study Population	Age Group (Year)	Notes	Intervention	Duration of Treatment	Tool for Asthma Diagnosis	Outcomes
Wood et al. [28], 2008	32 adults	Mean age of 52.1	Participants were on the low antioxidant diet for 10 days before the study commenced.	Tomato extract (45 mg lycopene/day) vs. tomato juice (45 mg lycopene/day) vs. placebo	3 × 7 day with a 10 days wash-out period between each treatment	Doctor-diagnosed asthma and having current (past 12 months) episodic respiratory symptoms	The LAO diet: ↓ %FEV1 ^a^ and %FVC, ↑ neutrophils increased both tomato juice and extract: ↓ airway neutrophil influx Neutrophils tomato extract: ↓ sputum neutrophil elastase activity
Baines et al. [29], 2009	10 adults diagnosed with stable asthma	Mean age of 63	No control group	The LAO diet ^b^	14 days	Doctor-diagnosed asthma and respiratory symptoms	The LAO diet: ↑ genes involved in the inflammatory and immune responses including the innate immune receptors TLR2, IL1R2, CD93, ANTXR2, the innate immune signalling molecules IRAK2, 3, MAP3K8 and neutrophil proteases.
Fogarty et al. [24], 2009	Intervention group n = 3233, Placebo group n = 3506	4–6	The control group received usual diet.	A daily piece of fruit (generally including apples, oranges or pears) adding to their usual diet	1 year	Questionnaire	↔
Wood et al. [30], 2012	137 adults	Mean age of 56	Participants randomized to the low vs. high antioxidant diet (5 servings of vegetables and 2 servings of fruit daily) for 14 days before the study commenced.	High antioxidant diet group received placebo, while, low antioxidant diet group received tomato extract (45 mg lycopene/day).	14 weeks	Doctor-diagnosed asthma and having current (past 12 months) episodic respiratory symptoms	The LAO diet: ↑ exacerbation, ↓ %FEV1 and %FVC
Lee et al. [60], 2013	192	10–12	The control group received placebos	“fruit and vegetable” capsule ^c^ + Fish oil capsules+ Probiotic capsules vs. placebo	16 weeks	Doctor-diagnosed asthma	The supplement group: ↑ FEV1, FVC and FEV1:FVC ratio, ↓ proportion of children using ICS
Garcia-Larsen et al. [25], 2014	32	6–10	Participants were randomly allocated to one of four groups. The control group received usual diet.	Having an apple or a banana or an apple + banana in addition to their normal diet	1 month	Respiratory tests	Groups 2 (adding banana) and 3 (adding banana + apple): ↓ levels of F_E_NO
Calatayud-Saez et al. [38], 2016	104 children with childhood asthma criteria for at least 1 year	1–5	No control group	Dietary re-education by a nutritional education programme named “Learning to Eat from the Mediterranean”	1 year	Doctor-diagnosed asthma	↓ The use of ICS

^a^ Abbreviation: F_E_NO, fractional exhaled nitric oxide; FEV1, forced expiratory volume in 1 s; FVC, forced vital capacity; ICS, inhaled corticosteroids; LAO, low antioxidant diet; ^b^ Included no more than one piece of fruit and two serves of vegetables per day and avoidance of tea, coffee, red wine, fruit juices, nuts, seeds, vitamin or mineral supplements and aspirin; ^c^ Contains 400 mg concentrate derived from grapes, plums, blueberries, raspberries, cranberries, cherries, cowberries, strawberries, artichokes, beets, carrots, broccoli, white cauliflower, kale, celery, and spinach.

## Supplementary Materials

The following are available online at http://www.mdpi.com/2072-6643/9/4/341/s1.
