# Supplementary material for: Effects of Fruit and Vegetable Consumption on Risk of Asthma, Wheezing and Immune Responses: A Systematic Review and Meta-Analysis
Source: Nutrients. 2017 Mar 29;9(4):341. doi: 10.3390/nu9040341 (PMC5409680; doi:10.3390/nu9040341)
Supplement: Supplementary file 1 [file nutrients-09-00341-s001.docx]

**Table E1- Additional characteristics of cross-sectional studies on the association between fruits and vegetables intakes and asthma**

| **Author (year)** | **Country** | **Adjustment applied** | **Study limitations** | **Study quality** |
| --- | --- | --- | --- | --- |
| Cook et al(1), 1997 | England | Age, height, gender, town, observer and room temperature | Reverse causality bias | + |
| La vecchia et al(2), 1998 | Italy | Gender, age, education, tobacco use, alcohol consumption | Lack of validation for the data collected, reverse causality bias | ᴓ |
| Forastiere et al(3), 2000 | Italy | Gender, study area, paternal education, household density, maternal smoking, paternal  smoking, dampness or mould in the child’s bedroom, parental asthma | Information bias | + |
| Priftanji et al(4), 2002 | Albania | age, gender, and history of cigarette smoking/ y | Reverse causality bias | + |
| Gilliland et al(5), 2003 | USA | Gender, age | Temporality and selection bias, reverse causality bias | ᴓ |
| Woods et al(6), 2003 | Australia | Age, gender, BMI, smoking status, region of birth, and family history of asthma | Reverse causality bias | + |
| Awasthi et al(7), 2004 | India | Age | Reverse causality bias, unmeasured confounders associated with life-style | + |
| Wong et al(8), 2004 | China | The propensity score, gender | Reverse causality bias | + |
| Lewis et al(9), 2005 | UK | Age, gender, and area | Reporting bias | + |
| Nja et al(10), 2005 | Norway | age, gender, area, parental education, parental atopy, exposure to dog and cat, and mothers’ smoking during  pregnancy and 0–11mo | recall bias, the age span of 10 y | + |
| Tabak et al(11), 2006 | Germany | Maternal education level, foreign descent, and total energy intake | Reverse causality bias | + |
| Cardinale et al(12), 2007 | Italy | gender,  height, weight, body mass index, area of residence, pater-  nal education, household crowding, parental smoking  habits, mold in the child’s room, and parental asthma | Reverse causality bias | + |
| Chatzi et al(13), 2007 | Greece | Age, gender, BMI, parental asthma, number of older siblings | Self-administered nature of the questionnaire, un-validated food frequency  questionnaire, reverse causality bias | ᴓ |
| Garcia-Marcos et al(14), 2007 | Spain | Gender, obesity, maternal smoking, siblings and exercise | Reverse causality bias | + |
| Okoko et al(15), 2007 | UK | Gender, age, Paracetamol/ Ibuprofen exposure group, vitamin, iron or other supplement use, ever lived on a farm, mould or mildew in house, finance source for home repairs, exposure to passive smoking, ethnic group, birth weight, breastfeeding, number of parents living with child, number of other children at home, parental education level | The authors could not adjust for some potential confounders, such as BMI and other foods and nutrients | + |
| Tsai et al(16), 2007 | Taiwan | Residential districts, gender, physician-diagnosed allergy | Reverse causality bias | + |
| Barros et al(17), 2008 | Portugal | Gender, education, age, total energy intake, BMI, physical activity score, smoking, atopy, rhinitis and inhaled corticosteroid (ICS) | Reverse causality bias | + |
| Castro-Rodriguez et al(18), 2008 | Spain | BMI, weight, gender, physical activity | Information bias, reverse causality bias | ᴓ |
| Chatzi et al(19), 2008 | Spain | Gender, parental asthma, maternal social class and education, BMI and total energy intake | Reverse causality bias | + |
| Garcia et al(20), 2008 | Colombia | Acetaminophen and antibiotic use, TV watching, maternal education, having cat at home | Recall bias, reverse causality bias | + |
| Takaoka et al(21), 2008 | Japan | age, parental asthma/allergy, smoking, all food items | Selection bias, information bias | ᴓ |
| Nagel et al(22), 2010 | 20 countries ^b^ | Gender, age, current exposure to tobacco smoke, number of siblings, parental atopic disease, exercise | Recall bias, the authors could not adjust for total energy intake and BMI | + |
| Arvaniti et al(23), 2011 | Greece | Age, gender, BMI, physical activity, energy | Recall bias and reverse causality bias | + |
| Lawson et al(24), 2011 | Canada | Age, gender, ethnicity, socioeconomic status | Information bias, missed data | ᴓ |
| Rosenlund et al(25), 2011 | Sweden | Gender, parental history of allergic disease, parental socioeconomic status, maternal smoking, maternal age at baseline, aged-adjusted BMI | Reverse causality bias | + |
| Rosenkranz et al(26), 2012 | Australia | Age, education, weight status, physical activity, smoking status | Selection bias, using self-reported instrument | ᴓ |
| Agrawal et al(27), 2013 | India | Gender, age, marital status, education, religion, employment status, wealth index, residence and geographic region | The measure of asthma prevalence was based on a single question | ᴓ |
| Ng et al(28), 2013 | Singapore | Gender, age height, smoking, occupational history, history of asthma and/or COPD, BMI physical activity | Using SQFFQ, reverse causality bias | + |
| Alphantonogeorgos et al(29), 2014 | Greece | Age, gender, overweight/ obesity, parental atopy, living environment (urban or rural) | Recall bias and reverse causality bias | + |
| Papadopoulou et al(30), 2014 | Greece | city of origin and exercise level, parental allergy history and breast feeding and other variables that showed significance in the univariate analyses (i.e., parental academic education, environmental indoor and outdoor factors) | Using SQFFQ, recall bias and reverse causality bias | + |
| Gomes de Luna Mde et al(31), 2015 | Brazil | Type of school, maternal education level, traffic  of trucks/bus, rhinitis, rhinoconjunctivitis, physical activity, previous use of paracetamol | Reverse causality bias | + |

^a^ **Abbreviation:** BMI, body mass index; COA, current occasional asthma; CSA, current severe asthma; ECRHS, the European community respiratory health survey screening questionnaire; FFQ, food frequency questionnaire, F&V, fruits and vegetables; ISAAC, international study of asthma and allergies in childhood questionnaire

^b^29 centres in 20 countries (ISAAC Phase II)

**Table E2- Additional characteristics of case-control studies on the association between fruits and vegetables intakes and asthma**

| **Author (year)** | **Country** | **Adjustment applied** | **Study limitations** | **Study quality** |
| --- | --- | --- | --- | --- |
| Hijazi et al(32), 2000 | Saudi Arabia | Gender, nationality, place of residence, mother's education, family history of asthma or hay fever, and presence of positive skin tests | Recall bias | + |
| Shaheen et al(33), 2001 | UK | Age, gender, BMI, social class, housing tenure, employment status, whether a single parent, smoking, passive smoke exposure at home, and total energy intake. | Incomplete information on age of onset of asthma | + |
| Patel et al(34), 2006 | UK | Pack years smoked, social class, BMI, increasing level of physical activity, and level of education | Cannot establish a causal relationship | + |
| Pastorino et al(35), 2006 | Brazil | Allergic mother, skin prick test, eczema, rhinitis, prematurity | Cannot reach causal relationship | + |
| Romieu et al(36), 2009 | Mexico | 5-day accumulated moving average O3 (ppb), 5-day accumulated average (maximum) PM2.5 (μg/m3), previous day minimum temperature, gender, body mass index, calories and chronological time | Small sample size of control children | + |
| Mendes et al(37), 2011 | Brazil | Neonatal period, age at onset of the disease, parental education, house hold income, presence of house hold pets, family history of asthma, personal history of allergic rhinitis, exposure to maternal smoking, exclusive breastfeeding, obesity, and regular consumption of fish, eggs in the last 30 days | Diagnosis and classification of asthma was based on only clinical history without lung function measures | ᴓ |
| Protudjer et al(38), 2012 | Canada | Breastfeeding, fast food consumption, weight, region of residence, family income, physical activity, and maternal history of asthma | Self-reported dietary data, selection bias, small sample size | + |
| Han et al(39), 2015 | USA | Age, gender, household income, parental asthma, body mass index, outdoor physical activity, early-life environmental tobacco smoke, and breastfeeding | Recall bias, social desirability bias, and inaccurate reporting were possible | ᴓ |

^a^ **Abbreviation:** BMI, body mass index; FFQ, food frequency questionnaire, F&V, fruits and vegetables; ISAAC, international study of asthma and allergies in childhood questionnaire; MDS, Mediterranean diet score; SQFFQ, semi-quantitative food frequency questionnaire.

**Table E3- Additional characteristics of cohort studies on the association between fruits and vegetables intakes and asthma**

| **Author (year)** | **Country** | **Adjustment applied** | **Study limitations** | **Study**  **quality** |
| --- | --- | --- | --- | --- |
| Butland et al(40), 1999 | UK | Gender, social class, smoking habit, salad/ raw vegetable intake | Lack of data on fruit type and portion size | + |
| Knekt et al(41), 2002 | Finland | Gender, age | Information on flavonoid intake may not be an accurate measure of the flavonoids available in the human body. | + |
| Farchi et al(42), 2003 | Italy | Gender, study area, paternal education, paternal smoking, paternal asthma, dampness or mald in child's room, household crowding | Information bias, reverse bias, the analysis considered symptoms and diet collected at the same time, the scope of the nutrients that were investigated is limited and information of total energy intake is unavailable | ᴓ |
| Romieu et al(43), 2006 | Mexico | age, total calorie intake, BMI, physical activity, smoking status, menopausal status, and the use of dietary supplements, smoking status | Definition of asthma was based on one question  regarding the occurrence of asthma attacks | + |
| Fitzsimon et al(44), 2007 | Ireland | Child’s birthweight, child’s gender, and smoke exposure in the home | Children's diet was not controlled | + |
| Willers et al(45), 2007 | Netherlands | Maternal age, paternal social class, maternal age of leaving full time education, maternal smoking during pregnancy, maternal asthma, maternal atopy, child’s birth weight, child’s sex, presence of older siblings, breast feeding and smoking in the child’s home at 5 years | Response bias | + |
| Chatzi et al(19), 2008 | Spain | Gender, parental asthma, maternal social class and education, BMI and total energy intake at age 6.5 years | Information bias | + |
| Willers et al(46), 2008 | Netherlands | Gender, maternal educational level, parental atopy, maternal smoking during pregnancy,  smoking in the house at age 8 years, breast feeding, presence of older siblings, birth weight, overweight mother, maternal  supplement use during pregnancy without folic acid and iron, region, and study arm (intervention study or natural history study) | Response bias | + |
| Bacopoulou et al(47), 2009 | Greece | Maternal residence, birth weight, gender, paternal occupation, maternal education parental marital status | High drop-out rates with a potential positive selection bias | ᴓ |
| Miyake et al(48), 2010 | Japan | Maternal age, gestation at baseline, residential municipality at baseline, family income, parental education, parental history of asthma, atopic eczema, and allergic rhinitis, changes in maternal diet in the previous 1 month, season when data at baseline were collected, maternal smoking during pregnancy, baby’s older siblings, baby’s gender, baby’s birth weight, household smoking in same room as infant, breastfeeding duration, and age of infant at third survey | The mother–child pairs in this study were likely not representative of Japanese mother–child pairs in the general population, the diet history questionnaire could only approximate consumption. | ᴓ |
| Uddenfeldt et al(49), 2010 | Sweden | Gender, heredity, hay fever, smoking, GI reflux, snoring, PA, building dampness, socioeconomic group, BMI | Life style factors were only reported on follow up, except for smoking, using self-reported data, asthma questionnaire was not validated for the elderly population | ᴓ |
| Nwaru et al(50), 2011 | Finland | gender, place of birth, season of birth, gestational age at birth, maternal age at birth, maternal basic education, maternal smoking during pregnancy, mode of delivery, number of siblings, parental asthma, parental allergic rhinitis, pets at home at 1 year of age, atopic eczema by 6 months of age | The difficulty of the FFQ to accurately quantify maternal antioxidant intake during pregnancy | + |
| Willers et al(51), 2011 | Netherlands | Gender, maternal educational level, parental atopy, maternal smoking during pregnancy, smoking in the house at 8 y of age, breast feeding, presence of older siblings, birth weight, overweight mother, overweight child at 8 y of age, geographical region and study arm (intervention or natural history) | The lack of data on consumption of specific types of fruit, and vegetables, use of self-reported dietary data, the FFQs were not validated in the study population | ᴓ |

^a^ **Abbreviation:** BMI, body mass index; DHQ, dietary habit questionnaire; FFQ, food frequency questionnaire, F&V, fruits and vegetables; ISAAC, international study of asthma and allergies in childhood questionnaire, SQFFQ, semi quantitative food frequency questionnaire

**Table E4- Additional characteristics of trials on the association between fruits and vegetables intakes and asthma**

| **Author (year)** | **Country** | **Adjustment applied** | **Study limitations** | **Study**  **quality** |
| --- | --- | --- | --- | --- |
| Wood et al(52), 2008 | Australia | - | The treatment phase was un-blinded, compliance with the low antioxidant diet deteriorated as the trial progressed | + |
| Baines et al(53), 2009 | Australia | - | Lacking a control group, not adjusted for confounding factors such as age, gender, inhaled steroid use, asthma severity, smoking, and atopic status for their relationship to airway gene expression, intra-individual differences in baseline antioxidant status may have an influence on airway gene expression | ᴓ |
| Fogarty et al(54), 2009 | UK | Age, gender, Townsend Score, exposure to smoking at home or baseline wheeze prevalence | Short duration of the intervention, limited consumption of the free daily fruit | + |
| Wood et al(55), 2012 | Australia | Gender, age, and smoking history | The treatment phase was un-blinded | + |
| Lee et al(56), 2013 | Taiwan | Age, gender, BMI, cluster effect (children’s school) and seasonal effect | Not using a factorial design to study the effects of the single items and the combined supplement | + |
| Garcia-Larsen et al(57), 2014 | UK | - | Small sample size, short duration | + |
| Calatayud-Saez et al(58), 2016 | Spain | Gender | Lack of randomized control group | ᴓ |

^a^ **Abbreviation:** FEV1, forced expiratory volume in 1 s; FVC, forced vital capacity; ICS, inhaled corticosteroids; LAO, low antioxidant diet;

^b^ Included no more than one piece of fruit and two serves of vegetables per day and avoidance of tea, coffee, red wine, fruit juices, nuts, seeds, vitamin or mineral supplements and aspirin.

^c^ Contains 400mg concentrate derived from grapes, plums, blueberries, raspberries, cranberries, cherries, cowberries, strawberries, artichokes, beets, carrots, broccoli, white cauliflower, kale, celery, spinach and tomatoes.

**References**

1. Cook DG, Carey IM, Whincup PH, Papacosta O, Chirico S, Bruckdorfer KR, et al. Effect of fresh fruit consumption on lung function and wheeze in children. Thorax. 1997 Jul;52(7):628-33. PubMed PMID: 9246135. Pubmed Central PMCID: PMC1758609. Epub 1997/07/01. eng.

2. La Vecchia C, Decarli A, Pagano R. Vegetable consumption and risk of chronic disease. Epidemiology (Cambridge, Mass). 1998 Mar;9(2):208-10. PubMed PMID: 9504293. Epub 1998/03/21. eng.

3. Forastiere F, Pistelli R, Sestini P, Fortes C, Renzoni E, Rusconi F, et al. Consumption of fresh fruit rich in vitamin C and wheezing symptoms in children. SIDRIA Collaborative Group, Italy (Italian Studies on Respiratory Disorders in Children and the Environment). Thorax. 2000 Apr;55(4):283-8. PubMed PMID: 10722767. Pubmed Central PMCID: PMC1745721. Epub 2000/03/18. eng.

4. Priftanji AV, Qirko E, Burr ML, Layzell JC, Williams KL. Factors associated with asthma in Albania. Allergy. 2002 Feb;57(2):123-8. PubMed PMID: 11929414. Epub 2002/04/04. eng.

5. Gilliland FD, Berhane KT, Li YF, Gauderman WJ, McConnell R, Peters J. Children's lung function and antioxidant vitamin, fruit, juice, and vegetable intake. Am J Epidemiol. 2003 Sep 15;158(6):576-84. PubMed PMID: 12965883. Epub 2003/09/11. eng.

6. Woods RK, Walters EH, Raven JM, Wolfe R, Ireland PD, Thien FC, et al. Food and nutrient intakes and asthma risk in young adults. Am J Clin Nutr. 2003 Sep;78(3):414-21. PubMed PMID: 12936923. Epub 2003/08/26. eng.

7. Awasthi S, Kalra E, Roy S, Awasthi S. Prevalence and risk factors of asthma and wheeze in school-going children in Lucknow, North India. Indian Pediatr. 2004 Dec;41(12):1205-10. PubMed PMID: 15623900. Epub 2004/12/30. eng.

8. Wong GW, Ko FW, Hui DS, Fok TF, Carr D, von Mutius E, et al. Factors associated with difference in prevalence of asthma in children from three cities in China: multicentre epidemiological survey. BMJ. 2004 Aug 28;329(7464):486. PubMed PMID: 15331473. Pubmed Central PMCID: PMC515199. Epub 2004/08/28. eng.

9. Lewis SA, Antoniak M, Venn AJ, Davies L, Goodwin A, Salfield N, et al. Secondhand smoke, dietary fruit intake, road traffic exposures, and the prevalence of asthma: a cross-sectional study in young children. Am J Epidemiol. 2005 Mar 1;161(5):406-11. PubMed PMID: 15718476. Epub 2005/02/19. eng.

10. Nja F, Nystad W, Lodrup Carlsen KC, Hetlevik O, Carlsen KH. Effects of early intake of fruit or vegetables in relation to later asthma and allergic sensitization in school-age children. Acta Paediatr. 2005 Feb;94(2):147-54. PubMed PMID: 15981746. Epub 2005/06/29. eng.

11. Tabak C, Wijga AH, de Meer G, Janssen NA, Brunekreef B, Smit HA. Diet and asthma in Dutch school children (ISAAC-2). Thorax. 2006 Dec;61(12):1048-53. PubMed PMID: 16244092. Pubmed Central PMCID: PMC2117046. Epub 2005/10/26. eng.

12. Cardinale F, Tesse R, Fucilli C, Loffredo MS, Iacoviello G, Chinellato I, et al. Correlation between exhaled nitric oxide and dietary consumption of fats and antioxidants in children with asthma. J Allergy Clin Immunol. 2007 May;119(5):1268-70. PubMed PMID: 17321576. Epub 2007/02/27. eng.

13. Chatzi L, Apostolaki G, Bibakis I, Skypala I, Bibaki-Liakou V, Tzanakis N, et al. Protective effect of fruits, vegetables and the Mediterranean diet on asthma and allergies among children in Crete. Thorax. 2007 Aug;62(8):677-83. PubMed PMID: 17412780. Pubmed Central PMCID: PMC2117278. Epub 2007/04/07. eng.

14. Garcia-Marcos L, Canflanca IM, Garrido JB, Varela AL, Garcia-Hernandez G, Guillen Grima F, et al. Relationship of asthma and rhinoconjunctivitis with obesity, exercise and Mediterranean diet in Spanish schoolchildren. Thorax. 2007 Jun;62(6):503-8. PubMed PMID: 17251311. Pubmed Central PMCID: PMC2117202. Epub 2007/01/26. eng.

15. Okoko BJ, Burney PG, Newson RB, Potts JF, Shaheen SO. Childhood asthma and fruit consumption. Eur Respir J. 2007 Jun;29(6):1161-8. PubMed PMID: 17301090. Epub 2007/02/16. eng.

16. Tsai HJ, Tsai AC. The association of diet with respiratory symptoms and asthma in schoolchildren in Taipei, Taiwan. J Asthma. 2007 Oct;44(8):599-603. PubMed PMID: 17943568. Epub 2007/10/19. eng.

17. Barros R, Moreira A, Fonseca J, de Oliveira JF, Delgado L, Castel-Branco MG, et al. Adherence to the Mediterranean diet and fresh fruit intake are associated with improved asthma control. Allergy. 2008 Jul;63(7):917-23. PubMed PMID: 18588559. Epub 2008/07/01. eng.

18. Castro-Rodriguez JA, Garcia-Marcos L, Alfonseda Rojas JD, Valverde-Molina J, Sanchez-Solis M. Mediterranean diet as a protective factor for wheezing in preschool children. J Pediatr. 2008 Jun;152(6):823-8, 8 e1-2. PubMed PMID: 18492525. Epub 2008/05/22. eng.

19. Chatzi L, Torrent M, Romieu I, Garcia-Esteban R, Ferrer C, Vioque J, et al. Mediterranean diet in pregnancy is protective for wheeze and atopy in childhood. Thorax. 2008 Jun;63(6):507-13. PubMed PMID: 18198206. Epub 2008/01/17. eng.

20. Garcia E, Aristizabal G, Vasquez C, Rodriguez-Martinez CE, Sarmiento OL, Satizabal CL. Prevalence of and factors associated with current asthma symptoms in school children aged 6-7 and 13-14 yr old in Bogota, Colombia. Pediatr Allergy Immunol. 2008 Jun;19(4):307-14. PubMed PMID: 18208464. Epub 2008/01/23. eng.

21. Takaoka M, Norback D. Diet among Japanese female university students and asthmatic symptoms, infections, pollen and furry pet allergy. Respir Med. 2008 Jul;102(7):1045-54. PubMed PMID: 18356034. Epub 2008/03/22. eng.

22. Nagel G, Weinmayr G, Kleiner A, Garcia-Marcos L, Strachan DP. Effect of diet on asthma and allergic sensitisation in the International Study on Allergies and Asthma in Childhood (ISAAC) Phase Two. Thorax. 2010 Jun;65(6):516-22. PubMed PMID: 20522849. Epub 2010/06/05. eng.

23. Arvaniti F, Priftis KN, Papadimitriou A, Papadopoulos M, Roma E, Kapsokefalou M, et al. Adherence to the Mediterranean type of diet is associated with lower prevalence of asthma symptoms, among 10-12 years old children: the PANACEA study. Pediatr Allergy Immunol. 2011 May;22(3):283-9. PubMed PMID: 21457335. Epub 2011/04/05. eng.

24. Lawson JA, Janssen I, Bruner MW, Madani K, Pickett W. Urban-rural differences in asthma prevalence among young people in Canada: the roles of health behaviors and obesity. Ann Allergy Asthma Immunol. 2011 Sep;107(3):220-8. PubMed PMID: 21875540. Epub 2011/08/31. eng.

25. Rosenlund H, Kull I, Pershagen G, Wolk A, Wickman M, Bergstrom A. Fruit and vegetable consumption in relation to allergy: disease-related modification of consumption? J Allergy Clin Immunol. 2011 May;127(5):1219-25. PubMed PMID: 21215999. Epub 2011/01/11. eng.

26. Rosenkranz RR, Rosenkranz SK, Neessen KJ. Dietary factors associated with lifetime asthma or hayfever diagnosis in Australian middle-aged and older adults: a cross-sectional study. Nutrition journal. 2012;11:84. PubMed PMID: 23057785. Pubmed Central PMCID: PMC3544658. Epub 2012/10/13. eng.

27. Agrawal S, Pearce N, Ebrahim S. Prevalence and risk factors for self-reported asthma in an adult Indian population: a cross-sectional survey. The international journal of tuberculosis and lung disease : the official journal of the International Union against Tuberculosis and Lung Disease. 2013 Feb;17(2):275-82. PubMed PMID: 23317966. Pubmed Central PMCID: PMC4284294. Epub 2013/01/16. eng.

28. Ng TP, Niti M, Yap KB, Tan WC. Dietary and supplemental antioxidant and anti-inflammatory nutrient intakes and pulmonary function. Public Health Nutr. 2014 Sep;17(9):2081-6. PubMed PMID: 24074036. Epub 2013/10/01. eng.

29. Alphantonogeorgos G, Panagiotakos DB, Grigoropoulou D, Yfanti K, Papoutsakis C, Papadimitriou A, et al. Investigating the associations between Mediterranean diet, physical activity and living environment with childhood asthma using path analysis. Endocr Metab Immune Disord Drug Targets. 2014;14(3):226-33. PubMed PMID: 25176184. Epub 2014/09/02. eng.

30. Papadopoulou A, Panagiotakos DB, Hatziagorou E, Antonogeorgos G, Matziou VN, Tsanakas JN, et al. Antioxidant foods consumption and childhood asthma and other allergic diseases: The Greek cohorts of the ISAAC II survey. Allergol Immunopathol (Madr). 2015 Jul-Aug;43(4):353-60. PubMed PMID: 25097022. Epub 2014/08/07. eng.

31. Gomes de Luna Mde F, Gomes de Luna JR, Fisher GB, de Almeida PC, Chiesa D, Carlos da Silva MG. Factors associated with asthma in adolescents in the city of Fortaleza, Brazil. J Asthma. 2015 Jun;52(5):485-91. PubMed PMID: 25405360. Epub 2014/11/19. eng.

32. Hijazi N, Abalkhail B, Seaton A. Diet and childhood asthma in a society in transition: a study in urban and rural Saudi Arabia. Thorax. 2000 Sep;55(9):775-9. PubMed PMID: 10950897. Pubmed Central PMCID: PMC1745853. Epub 2000/08/19. eng.

33. Shaheen S, Sterne JC, Thompson R, Songhurst C, Margetts B, Burney PJ. Dietary Antioxidants and Asthma in Adults. Am J Respir Crit Care Med. 2001 2001/11/15;164(10):1823-8.

34. Patel BD, Welch AA, Bingham SA, Luben RN, Day NE, Khaw KT, et al. Dietary antioxidants and asthma in adults. Thorax. 2006 May;61(5):388-93. PubMed PMID: 16467075. Pubmed Central PMCID: PMC2111195. Epub 2006/02/10. eng.

35. Pastorino AC, Rimazza RD, Leone C, Castro AP, Sole D, Jacob CM. Risk factors for asthma in adolescents in a large urban region of Brazil. J Asthma. 2006 Nov;43(9):695-700. PubMed PMID: 17092851. Epub 2006/11/10. eng.

36. Romieu I, Barraza-Villarreal A, Escamilla-Nunez C, Texcalac-Sangrador JL, Hernandez-Cadena L, Diaz-Sanchez D, et al. Dietary intake, lung function and airway inflammation in Mexico City school children exposed to air pollutants. Respir Res. 2009;10:122. PubMed PMID: 20003306. Pubmed Central PMCID: PMC2806363. Epub 2009/12/17. eng.

37. Mendes AP, Zhang L, Prietsch SO, Franco OS, Gonzales KP, Fabris AG, et al. Factors associated with asthma severity in children: a case-control study. J Asthma. 2011 Apr;48(3):235-40. PubMed PMID: 21332431. Epub 2011/02/22. eng.

38. Protudjer JL, Sevenhuysen GP, Ramsey CD, Kozyrskyj AL, Becker AB. Low vegetable intake is associated with allergic asthma and moderate-to-severe airway hyperresponsiveness. Pediatric pulmonology. 2012 Dec;47(12):1159-69. PubMed PMID: 22628152. Epub 2012/05/26. eng.

39. Han YY, Forno E, Brehm JM, Acosta-Perez E, Alvarez M, Colon-Semidey A, et al. Diet, interleukin-17, and childhood asthma in Puerto Ricans. Ann Allergy Asthma Immunol. 2015 Oct;115(4):288-93 e1. PubMed PMID: 26319606. Pubmed Central PMCID: PMC4721241. Epub 2015/09/01. eng.

40. Butland BK, Strachan DP, Anderson HR. Fresh fruit intake and asthma symptoms in young British adults: confounding or effect modification by smoking? Eur Respir J. 1999 Apr;13(4):744-50. PubMed PMID: 10362034. Epub 1999/06/11. eng.

41. Knekt P, Kumpulainen J, Jarvinen R, Rissanen H, Heliovaara M, Reunanen A, et al. Flavonoid intake and risk of chronic diseases. Am J Clin Nutr. 2002 Sep;76(3):560-8. PubMed PMID: 12198000. Epub 2002/08/29. eng.

42. Farchi S, Forastiere F, Agabiti N, Corbo G, Pistelli R, Fortes C, et al. Dietary factors associated with wheezing and allergic rhinitis in children. Eur Respir J. 2003 Nov;22(5):772-80. PubMed PMID: 14621084. Epub 2003/11/19. eng.

43. Romieu I, Varraso R, Avenel V, Leynaert B, Kauffmann F, Clavel-Chapelon F. Fruit and vegetable intakes and asthma in the E3N study. Thorax. 2006 Mar;61(3):209-15. PubMed PMID: 16396945. Pubmed Central PMCID: PMC1974844. Epub 2006/01/07. eng.

44. Fitzsimon N, Fallon U, O'Mahony D, Loftus BG, Bury G, Murphy AW, et al. Mothers' dietary patterns during pregnancy and risk of asthma symptoms in children at 3 years. Ir Med J. 2007 Sep;100(8):suppl 27-32. PubMed PMID: 17955698. Epub 2007/10/25. eng.

45. Willers SM, Devereux G, Craig LC, McNeill G, Wijga AH, Abou El-Magd W, et al. Maternal food consumption during pregnancy and asthma, respiratory and atopic symptoms in 5-year-old children. Thorax. 2007 Sep;62(9):773-9. PubMed PMID: 17389754. Pubmed Central PMCID: PMC2117307. Epub 2007/03/29. eng.

46. Willers SM, Wijga AH, Brunekreef B, Kerkhof M, Gerritsen J, Hoekstra MO, et al. Maternal food consumption during pregnancy and the longitudinal development of childhood asthma. Am J Respir Crit Care Med. 2008;178(2):124-31 8p. PubMed PMID: 105813081. Language: English. Entry Date: 20080912. Revision Date: 20150711. Publication Type: Journal Article.

47. Bacopoulou F, Veltsista A, Vassi I, Gika A, Lekea V, Priftis K, et al. Can we be optimistic about asthma in childhood? A Greek cohort study. J Asthma. 2009 Mar;46(2):171-4. PubMed PMID: 19253125. Epub 2009/03/03. eng.

48. Miyake Y, Sasaki S, Tanaka K, Hirota Y. Consumption of vegetables, fruit, and antioxidants during pregnancy and wheeze and eczema in infants. Allergy. 2010 Jun 1;65(6):758-65. PubMed PMID: 20102358. Epub 2010/01/28. eng.

49. Uddenfeldt M, Janson C, Lampa E, Leander M, Norbäck D, Larsson L, et al. High BMI is related to higher incidence of asthma, while a fish and fruit diet is related to a lower–: Results from a long-term follow-up study of three age groups in Sweden. Respiratory Medicine. 2010 7//;104(7):972-80.

50. Nwaru BI, Erkkola M, Ahonen S, Kaila M, Kronberg-Kippila C, Ilonen J, et al. Intake of antioxidants during pregnancy and the risk of allergies and asthma in the offspring. Eur J Clin Nutr. 2011 Aug;65(8):937-43. PubMed PMID: 21559033. Epub 2011/05/12. eng.

51. Willers SM, Wijga AH, Brunekreef B, Scholtens S, Postma DS, Kerkhof M, et al. Childhood diet and asthma and atopy at 8 years of age: the PIAMA birth cohort study. Eur Respir J. 2011 May;37(5):1060-7. PubMed PMID: 21109553. Epub 2010/11/27. eng.

52. Wood LG, Garg ML, Powell H, Gibson PG. Lycopene-rich treatments modify noneosinophilic airway inflammation in asthma: proof of concept. Free Radic Res. 2008 Jan;42(1):94-102. PubMed PMID: 18324527. Epub 2008/03/08. eng.

53. Baines KJ, Wood LG, Gibson PG. The nutrigenomics of asthma: molecular mechanisms of airway neutrophilia following dietary antioxidant withdrawal. OMICS. 2009 Oct;13(5):355-65. PubMed PMID: 19715394. Epub 2009/09/01. eng.

54. Fogarty AW, Antoniak M, Venn AJ, Davies L, Goodwin A, Salfield N, et al. A natural experiment on the impact of fruit supplementation on asthma symptoms in children. Eur Respir J. 2009 Mar;33(3):481-5. PubMed PMID: 19213783.

55. Wood LG, Garg ML, Smart JM, Scott HA, Barker D, Gibson PG. Manipulating antioxidant intake in asthma: a randomized controlled trial. Am J Clin Nutr. 2012 Sep;96(3):534-43. PubMed PMID: 22854412. Epub 2012/08/03. eng.

56. Lee SC, Yang YH, Chuang SY, Huang SY, Pan WH. Reduced medication use and improved pulmonary function with supplements containing vegetable and fruit concentrate, fish oil and probiotics in asthmatic school children: a randomised controlled trial. Br J Nutr. 2013 Jul 14;110(1):145-55. PubMed PMID: 23211647. Epub 2012/12/06. eng.

57. Garcia-Larsen V, Bush A, Boyle RJ, Shaheen SO, Warner JO, Athersuch T, et al. O06-The Chelsea, asthma and fresh fruit intake in children (CHAFFINCH) trial-pilot study. Clinical and Translational Allergy [Internet]. 2014; 4:[6dummy p.]. Available from: <http://onlinelibrary.wiley.com/o/cochrane/clcentral/articles/999/CN-01052999/frame.html>.

58. Calatayud-Saez FM, Calatayud Moscoso Del Prado B, Gallego Fernandez-Pacheco JG, Gonzalez-Martin C, Alguacil Merino LF. Mediterranean diet and childhood asthma. Allergol Immunopathol (Madr). 2016 Mar-Apr;44(2):99-105. PubMed PMID: 26278484. Epub 2015/08/19. eng.
